# The expression landscape and heterogeneity of nectin-4, HER2, and Trop-2 in urothelial carcinoma: clinicopathological correlations and implications for ADC-guided precision therapy

**DOI:** 10.3389/fonc.2026.1887794

**Published:** 2026-07-08

**Authors:** Shaobo Qin, Zhicheng Dai, Zhenxing Hu, Xiaohui Wang, Huiyan Zhang, Haotian Yang, Jie Liu, Lianghong Ma

**Affiliations:** 1 Department of Clinical Medicine, Shandong Second Medical University, Weifang, China; 2 The First Clinical Medical College, Ningxia Medical University, Yinchuan, China; 3 Department of Pain Treatment, Weifang People’s Hospital, Weifang, China; 4 Department of Radiology, General Hospital of Ningxia Medical University, Yinchuan, China; 5 Department of Urology, Linyi People’s Hospital, Linyi, China; 6 Department of Urology, General Hospital of Ningxia Medical University, Yinchuan, China

**Keywords:** antibody-drug conjugates, co-expression, HER2, heterogeneity, nectin-4, Trop-2, urothelial carcinoma

## Abstract

**Background:**

Antibody-drug conjugates (ADCs) have redefined the treatment of urothelial carcinoma (UC). However, the spatiotemporal heterogeneity and co-expression landscapes of these targets across diverse anatomical sites and pathological stages remain poorly understood. This study aimed to map these patterns and explore their clinical implications for personalized ADC therapy.

**Methods:**

A cohort of 118 UC specimens was analyzed, including matched pairs of primary tumors with adjacent normal tissues (n=60), carcinoma *in situ* (CIS, n=8), and lymph node (LN) metastases (n=12). Expression levels were evaluated via immunohistochemistry (IHC). Statistical analyses included the Wilcoxon signed-rank test, Mann–Whitney U test, and multivariable logistic regression. A novel Δscore (tumor score - normal score) was introduced to quantify target “net gain.”

**Results:**

High expression (IHC 2+ or higher) of at least one target occurred in 90.7 percent of patients, with 56.8 percent showing multi-target co-expression. HER2 and Trop-2 were significantly upregulated during the transition from CIS to invasive carcinoma (P = 0.034 and P = 0.039). While primary tumors and metastases showed global stability (P greater than 0.05), Nectin-4 tended to downregulate in metastases (negative ranks in 50 percent of pairs). Trop-2 expression was significantly higher in the lower than the upper urinary tract (P = 0.042), whereas HER2 and Nectin-4 were uniformly distributed. Furthermore, a high Δ-Trop-2 was independently associated with muscle invasion, lymphovascular invasion, and metastasis.

**Conclusions:**

In the “post-EV+P” era, our findings reveal significant antigenic drift and anatomical expression biases during disease progression, advocating for dynamic, multi-site molecular profiling over static primary-site assessment. This provides a translational blueprint to optimize ADC sequencing and overcome heterogeneity-driven resistance. Finally, we introduce the novel Δscore as a new metric for further testing in quantifying net antigenic gain and predicting aggressive phenotypes.

## 1 Introduction

Urothelial carcinoma is one of the most prevalent epithelial malignancies of the urinary system that can originate throughout the urinary tract, including the bladder, renal pelvis, ureter, and urethra (1). Marked by pronounced biological heterogeneity and clinical diversity, UC presents a major public health challenge due to its substantial global disease burden. Long-term management remains formidable, driven by high recurrence rates, a propensity for muscle-invasive progression, and dismal clinical outcomes following metastatic transition.

The current stage-dependent therapeutic paradigm relies on surgery, perioperative regimens, and systemic therapies (2). For advanced UC, although platinum-based chemotherapy and immune checkpoint inhibitors have improved clinical outcomes, therapeutic responses remain markedly heterogeneous, and many “platinum-ineligible” patients cannot tolerate standard regimens due to renal impairment or poor performance status. Persistent resistance, post-treatment relapse, and limited response durations present a critical unmet clinical need. In this context, Antibody-Drug Conjugates (ADCs) have emerged as a transformative class of targeted agents.

ADCs are highly precise targeted antitumor agents consisting of three core components: a monoclonal antibody, a chemical linker, and a cytotoxic payload. By utilizing the antibody as a vehicle conjugated to biologically active cytotoxic molecules, ADCs selectively target cell-surface antigens to deliver potent payloads directly into tumor tissues, effectively combining the precision of targeted therapy with the efficacy of systemic chemotherapy.In recent years, ADCs have rapidly transformed the therapeutic landscape of urothelial carcinoma. Following the landmark results of the Phase 3 EV-302 (KEYNOTE-A39) trial (3), enfortumab vedotin (EV)—a Nectin-4-directed ADC—has achieved unprecedented survival benefits in advanced UC. Its combination with pembrolizumab has subsequently been established as the new global standard for first-line treatment (4). Furthermore, other clinical trials, such as DESTINY-PanTumor02 (5) and the RC48-C005/C009 studies (6), have highlighted the significant potential of ADCs targeting HER2 in the management of UC.

However, ADC therapy is not “universally efficacious”; the degree of clinical benefit varies significantly across different molecular targets, pharmacological designs, and patient populations. A notable case is sacituzumab govitecan (SG), a Trop-2-directed ADC that initially received accelerated approval but later faced a regulatory setback when its indication for advanced UC was withdrawn in the United States (7). This clinical pivot necessitates a critical reflection: does the widespread expression of a target antigen inherently guarantee robust therapeutic benefit in the context of the complex heterogeneity of UC? The challenges encountered with SG may not stem from the failure of the target itself, but rather suggest that, in the absence of biomarker-driven patient selection, intratumoral heterogeneity and antigen downregulation during metastatic progression may lead to treatment failure (8). Consequently, a systematic assessment of the true “expression landscape” of these targets across various anatomical sites and pathological stages is imperative to refine the precision of ADC-guided therapy.

Among key targets, Nectin-4 expression stability dictates the reach of first-line EV benefits, while Trop-2 maintains therapeutic potential due to its high baseline prevalence despite setbacks. Conversely, HER2 exhibits pronounced variability fueling precision exploration with agents like disitamab vedotin (RC48).

In view of these considerations, the present study sought to systematically evaluate the expression profiles of Nectin-4, Trop-2, and HER2 in a cohort of 118 patients with UC using IHC. Beyond assessing expression abundance across various anatomical sites, we innovatively investigated expression shifts across complex pathological scenarios, including malignant versus adjacent tissues, primary versus metastatic lesions, and CIS versus invasive components. Furthermore, the correlations between these target profiles and clinicopathological characteristics were explored. By integrating the protein expression landscapes of these three key targets, this study aims to provide a robust pathological basis for optimizing precision treatment sequences in the “post-EV+P era,” ultimately offering translational insights for identifying patients most likely to benefit from ADCs and establishing stratified therapeutic paradigms for UC.

## 2 Materials and methods

### 2.1 Patients and tissue samples

In this retrospective study, tissue microarrays (TMAs) were constructed using specimens from 118 patients with primary urothelial carcinoma originating from diverse anatomical sites. All patients underwent surgical resection at Linyi People’s Hospital (Shandong, China) between January 2024 and December 2025. The cohort comprised 17 cases of upper tract urothelial carcinoma (UTUC; including 12 renal pelvis and 5 ureteral cancers) and 101 cases of lower tract UC (including 99 bladder and 2 urethral cancers). Within this cohort, 12 patients presented with lymph node metastases, 8 with concurrent carcinoma *in situ*, and 2 with distant metastases. Immunohistochemical staining was performed on the 118 primary tumor tissues, as well as all identified positive lymph node metastases, CIS lesions, and distant metastatic tissues. Furthermore, 60 matched adjacent normal tissues (ANT) were included as internal controls to evaluate the expression of Nectin-4, Trop-2, and HER2. This study was conducted in accordance with the ethical standards of the Declaration of Helsinki and was approved by the Institutional Review Board of Linyi People’s Hospital (Protocol No.: 202503-H-011). Written informed consent was obtained from all participants prior to TMA construction. Clinicopathological data were retrieved from electronic medical records and follow-up databases. Detailed clinical characteristics of the study population are summarized in **Table 1**.

**Table 1:** Patient characteristics.

Variable Value	Value
Age (year), mean ± SD	68 ± 10
Sex, n (%)	
Male	96 (81%)
Female	22 (19%)
Primary site, n (%)	
Renal pelvis	12 (10%)
Ureter	5 (4%)
Bladder	99 (84%)
Urethra	2 (2%)
Pathology, n (%)	
T-Stating	
Ta	41 (35%)
T1	40 (34%)
T2	13 (11%)
T3	18 (15%)
4	6 (5%)
R-Stating	
Rx	
R0	116 (98%)
R1	2 (2%)
Tumor Grade, n (%)	
Low-grade	39 (33%)
High-grade	79 (67%)
Tumor thrombus, n (%)	
Yes	17 (14%)
No	101 (86%)
Carcinoma *in situ*, n (%)	
Yes	9 (8%)
No	109 (82%)
Recurrence, n (%)	
Yes	8 (7%)
No	110 (93%)
Metastasis, n (%)	
No	104 (88%)
Yes, Lymph node metastasis	12 (10%)
Yes, Distant metastasis	2 (2%)
Muscle invasion, n (%)	
Yes	37 (31%)
No	81 (69%)

### 2.2 Specimen selection and clinicopathological variables

Representative tumor regions were identified based on hematoxylin and eosin (H&E)-stained sections for IHC analysis. Priority was given to areas with high tumor cellularity and minimal necrosis to ensure optimal antigen detection. For patients presenting with concurrent CIS or metastatic lesions, these components were evaluated and scored alongside the primary tumor. The following clinicopathological variables were extracted: age, sex, primary tumor location (renal pelvis, ureter, bladder, or urethra; further categorized as UTUC vs. lower tract UC), tumor size (maximum diameter), pathological T (pT) stage, lymph node status (pN stage), clinical stage, histological grade (WHO 2022 classification), presence of concurrent CIS, lymphovascular invasion, and recurrence status.

### 2.3 Immunohistochemistry

Formalin-fixed paraffin-embedded (FFPE) tissues were sectioned at a thickness of approximately 4 μm. Following standard deparaffinization and rehydration, heat-induced epitope retrieval (HIER) was performed using EDTA buffer (pH 9.0). Endogenous peroxidase activity was quenched, and non-specific binding was blocked. The sections were then incubated with primary antibodies against HER2 (1:500; 60311-1-Ig; Proteintech, Wuhan, China), Trop-2 (1:500; 27360-1-AP; Proteintech, Wuhan, China), and Nectin-4 (1:200; 21903-1-AP; Proteintech, Wuhan, China) overnight at 4 °C. Subsequently, the slides were incubated with HRP-conjugated goat anti-rabbit/mouse secondary antibodies for 30 minutes at 37 °C. Immunoreactivity was visualized using freshly prepared 3,3’-diaminobenzidine (DAB) chromogen. The sections were then counterstained with hematoxylin, dehydrated, and coverslipped. For each staining batch, known high-expressing UC tissues were utilized as positive controls, while the primary antibody was replaced with phosphate-buffered saline (PBS) for negative controls to ensure experimental specificity.

### 2.4 Slide interpretation and scoring

All IHC-stained sections were independently evaluated by two board-certified pathologists blinded to the clinicopathological data. In cases of discrepancy, a consensus was reached through simultaneous re-evaluation, with arbitration by a senior pathologist if necessary. HER2 expression was assessed according to the ASCO/CAP Gastric Cancer scoring criteria, the established standard for non-breast solid tumors exhibiting basolateral or lateral staining patterns (5, 9). Scoring was defined as follows: score 0, no reactivity or membranous reactivity in < 10% of tumor cells; score 1+, faint/barely perceptible membranous reactivity in ≥ 10% of tumor cells, with cells reactive only in part of their membrane; score 2+, weak to moderate complete, basolateral, or lateral membranous reactivity in ≥ 10% of tumor cells; and score 3+, strong complete, basolateral, or lateral membranous reactivity in ≥ 10% of tumor cells. For Trop-2 and Nectin-4, a semi-quantitative H-score was calculated using the formula: H-score = (1 *P1) + (2*P2) + (3*P3), where P1–P3 represent the percentage of positive cells at each intensity (range 0–300). Specimens were subsequently classified into four tiers: negative (score 0, H-score 0–14), low (score 1+, H-score 15–99), moderate (score 2+, H-score 100–199), and high (score 3+, H-score 200–300) (10, 11). For binary classification, “low expression” and “high expression” were defined as scores 0–1+ and 2+–3+, respectively. Notably, in alignment with the clinical eligibility criteria of the RC48-C005 and RC48-C009 trials (6), HER2 “high expression” in this study was defined as an IHC score of ≥ 2 +. This rationale is based on the mechanism of ADCs, which utilize target proteins as molecular anchors for cytotoxic payload delivery rather than relying solely on oncogenic driver inhibition (8). Consequently, fluorescence *in situ* hybridization (FISH) status was not assessed. Adjacent normal tissues, metastatic lesions, and CIS components were evaluated using the same standardized scoring system.

### 2.5 Statistical analysis

Statistical analyses were performed using SPSS software (version 29.0; IBM Corp., Armonk, NY, USA), and data visualization was conducted with GraphPad Prism (version 8.0; GraphPad Software, San Diego, CA, USA). Continuous variables are presented as mean ± standard deviation (SD) or median [interquartile range (IQR)], as appropriate. Categorical variables are reported as frequencies and percentages. A two-tailed P-value < 0.05 was considered statistically significant, while a P-value < 0.1 was interpreted as indicating a statistical trend.

For paired comparisons (e.g., primary tumor vs. matched adjacent normal tissue), the Wilcoxon signed-rank test was utilized for ordinal data (H-scores/IHC tiers), and McNemar’s test was employed for dichotomized binary data. For independent two-group comparisons (e.g., UTUC vs. lower tract UC; clinicopathological subgroups), the Mann–Whitney U test and the Chi-square test (or Fisher’s exact test, where appropriate) were used for ordinal and categorical variables, respectively. Multiple group comparisons across anatomical sites were analyzed using the Kruskal–Wallis H test, followed by *post-hoc* pairwise comparisons with Bonferroni correction for significant findings.

Correlations between target expression levels and clinicopathological variables were evaluated using Spearman’s rank correlation analysis. To identify predictors of IHC expression, binary logistic regression was performed for “high expression” outcomes, with results reported as odds ratios (ORs) and 95% confidence intervals (CIs). For ordinal outcomes (0–3+ tiers), ordinal logistic regression was employed after verifying the proportional odds assumption via the test of parallel lines. Covariates were selected based on clinical relevance and univariate analysis findings, with careful consideration to avoid model overfitting.

## 3 Results

**Figure 1:**
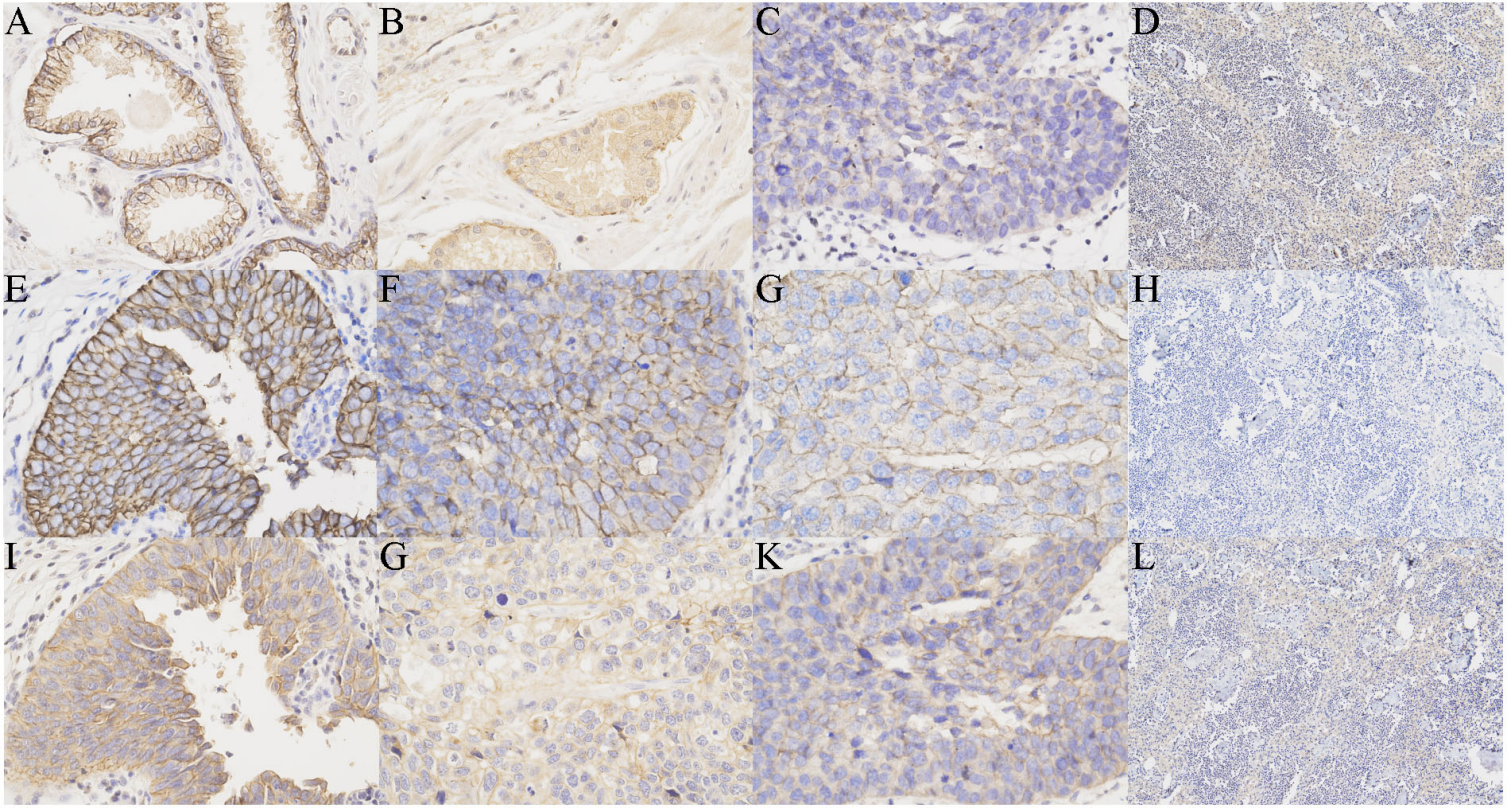
Immunohistochemical analysis of Nectin-4, Trop-2, and HER2 expression in urothelial carcinoma specimens. Representative images illustrate the spectrum of staining intensities observed across tissue samples. **(A–D)** Nectin-4 expression: **(A)** high (3+, ×400), **(B)** moderate (2+, ×400), **(C)** low (1+, ×400), and **(D)** negative (0, ×100). **(E–H)** Trop-2 expression: **(E)** high (3+, ×400), **(F)** moderate (2+, ×400), **(G)** low (1+, ×400), and **(H)** negative (0, ×100). **(I–L)** HER2 expression: **(I)** high (3+, ×400), **(J)** moderate (2+, ×400), **(K)** low (1+, ×400), and **(L)** negative (0, ×100).

**Figure 2:**
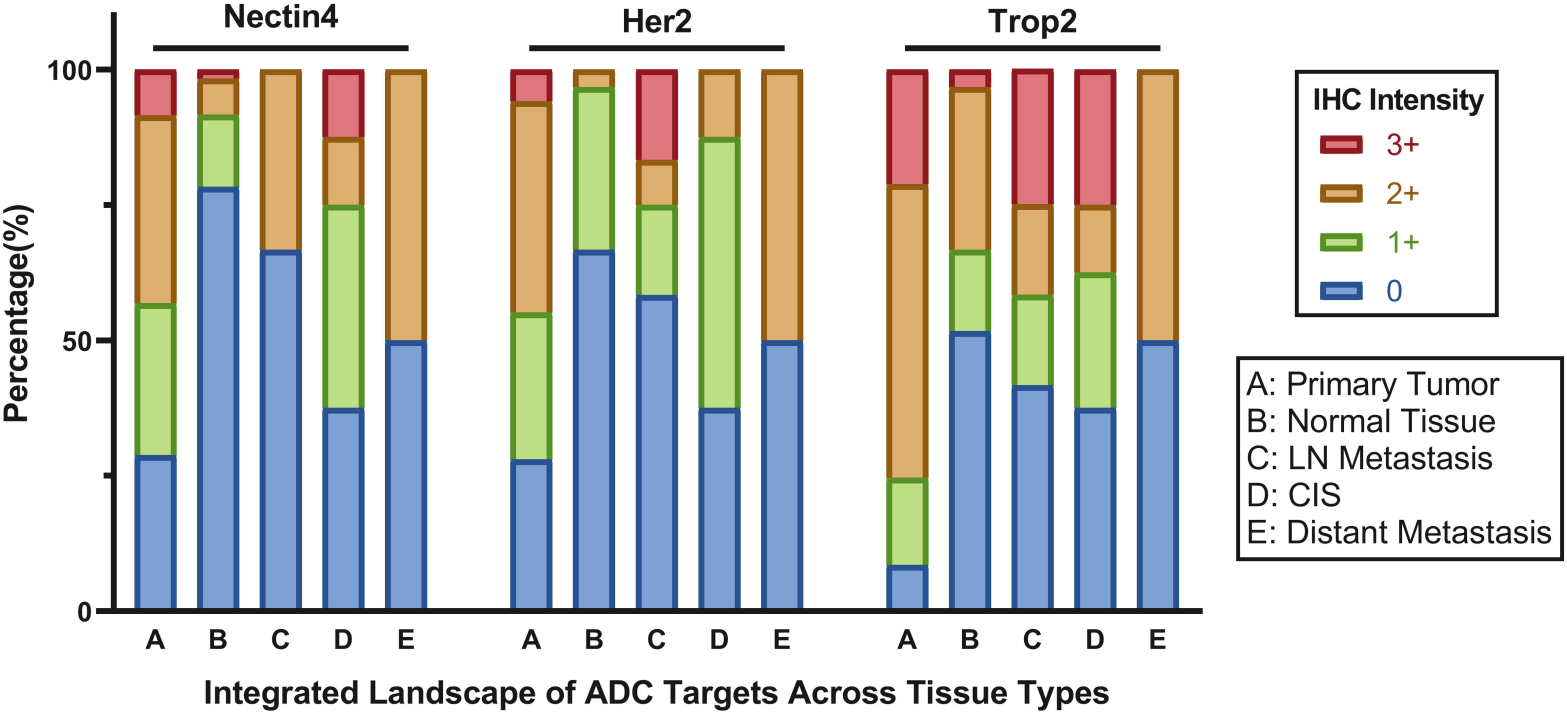
Integrated expression landscape of ADC targets across diverse tissue types in urothelial carcinoma. Stacked bar charts illustrate the distribution of immunohistochemical staining intensities (0, 1+, 2+, and 3+) for each target across different pathological contexts. The y-axis represents the percentage of cases within each intensity category for the specified tissue type.

Immunoreactivity for HER2 was predominantly localized to the cell membrane of tumor cells, exhibiting annular, semi-annular, or basolateral staining patterns with a color spectrum ranging from pale yellow to dark brown (9). Notably, HER2 expression was virtually absent in normal urothelial and lymphoid tissues (**Figure 1**). Trop-2 and Nectin-4 also displayed primary membranous expressions, with occasional cytoplasmic staining observed. Both targets showed negligible or low-level expression in normal urothelial and lymphoid tissues (**Figure 1**). Trop-2 was characterized by robust and intense membranous staining across most positive cases (12). As a cell-cell adhesion molecule, Nectin-4 staining was concentrated at intercellular junctions, exhibiting prominent membranous accentuation and occasional granular cytoplasmic patterns within the tumor tissues (12). The integrated landscape of ADC targets across various tissue types is illustrated in **Figure 2**. The detailed distribution and IHC staining characteristics of these three targets are summarized in **Table 2**.

**Table 2:** IHC staining results.

Target/score	Primary tumor (n=118)	Normal tissue (n=60)	LN metastasis (n=12)	CIS (n=8)	Distant metastasis (n=2)
Her2					
0 (Negative)	33 (28.0%)	40 (66.7%)	7 (58.3%)	3 (37.5%)	1 (50.0%)
1+ (Low)	32 (27.1%)	18 (30.0%)	2 (16.7%)	4 (50.0%)	0 (0.0%)
2+ (Medium)	46 (39.0%)	2 (3.3%)	1 (8.3%)	1 (12.5%)	1 (50.0%)
3+ (High)	7 (5.9%)	0 (0.0%)	2 (16.7%)	0 (0.0%)	0 (0.0%)
Total Positive (2+/3+)	44.90%	3.30%	25.00%	12.50%	50.00%
Trop2					
0 (Negative)	10 (8.5%)	31 (51.7%)	5 (41.7%)	3 (37.5%)	1 (50.0%)
1+ (Low)	19 (16.1%)	9 (15.0%)	2 (16.7%)	2 (25.0%)	0 (0.0%)
2+ (Medium)	64 (54.2%)	18 (30.0%)	2 (16.7%)	1 (12.5%)	1 (50.0%)
3+ (High)	25 (21.2%)	2 (3.3%)	3 (25.0%)	2 (25.0%)	0 (0.0%)
Total Positive (Any+)	91.50%	48.30%	58.30%	62.50%	50.00%
Nectin-4					
0 (Negative)	34 (28.8%)	47 (78.3%)	8 (66.7%)	3 (37.5%)	1 (50.0%)
1+ (Low)	33 (28.0%)	8 (13.3%)	0 (0.0%)	3 (37.5%)	0 (0.0%)
2+ (Medium)	41 (34.7%)	4 (6.7%)	4 (33.3%)	1 (12.5%)	1 (50.0%)
3+ (High)	10 (8.5%)	1 (1.7%)	0 (0.0%)	1 (12.5%)	0 (0.0%)
Total Positive (Any+)	71.20%	21.70%	33.30%	62.50%	50.00%

Data are presented as n (%). IHC score: 0, negative, 1+, weak, 2+, moderate, 3+, strong. CIS, carcinoma *in situ*; LN = lymph node.

The results of this study were categorized into primary and secondary outcomes.

### 3.1 Primary outcomes

#### 3.1.1 Differential expression between primary tumor and matched adjacent normal tissues

**Figure 3:**
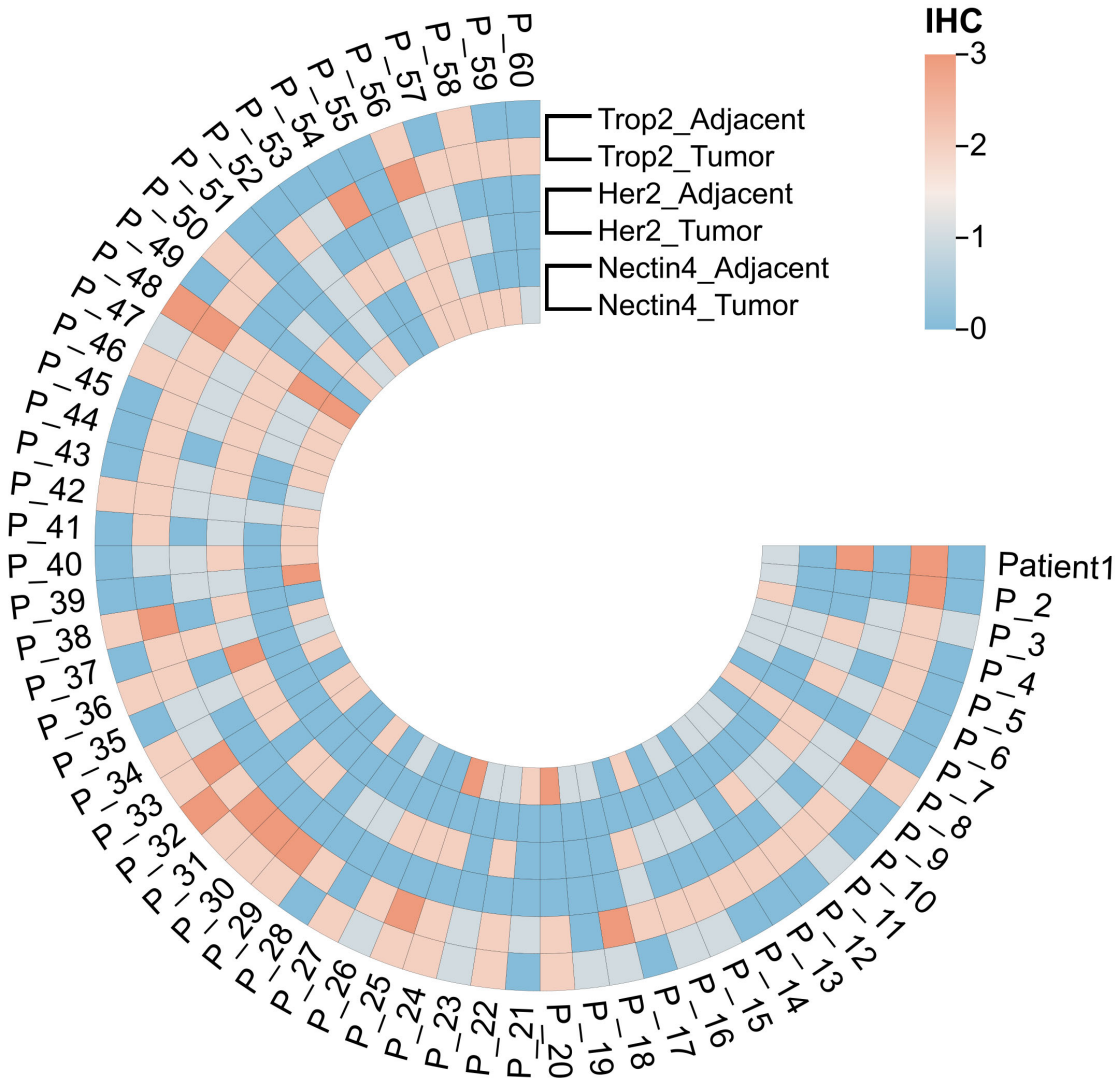
Circular heatmap illustrating the paired expression patterns and immunohistochemical intensity scores of ADC targets in tumor and adjacent normal tissues across 60 patients with matched specimens. Each radial sector represents an individual patient, chronologically arranged clockwise from Patient 1 to P_60 based on their sample collection IDs. The concentric rings are organized in pairs for each target: Trop-2, HER2, and Nectin-4. The color gradient represents the IHC intensity scores, ranging from blue (0, negative) to orange-red (3, high expression). The brackets on the right side indicate the paired alignment of primary tumor and matched adjacent normal tissue layers for each respective target.

In 60 matched pairs of specimens, the IHC expression intensities of HER2, Trop-2, and Nectin-4 were significantly higher in primary tumor tissues than in matched adjacent normal tissues (Wilcoxon signed-rank test: HER2, Z = -5.419; Trop-2, Z = -5.363; Nectin-4, Z = -5.531; all P < 0.001). These results underscore the high tumor-specificity of these three targets within the urinary tract. Representative IHC staining patterns showing the contrast between malignant and benign tissues are presented in **Figure 3**, and the quantitative comparisons are summarized in **Table 3**.

**Table 3:** Paired comparisons between tumor and adjacent normal tissues.

Marker	N	Intensity shift* (Normal<tumor/normal>tumor/tie)	Wilcoxon Z	Wilcoxon P	Effect size r^†^
HER2	60	39/2/19	-5.419	**<0.001**	0.700
Trop2	60	43/4/13	-5.363	**<0.001**	0.692
Nectin4	60	38/0/22	-5.531	**<0.001**	0.714

*Intensity shift shows paired direction based on Wilcoxon ranks (Normal<Tumor indicates higher intensity in tumor).

^†^Effect size r = |Z|/√n.

Bold values indicate statistical significance (p < 0.05).

#### 3.1.2 Expression heterogeneity across diverse pathological scenarios

To evaluate the spatio-temporal stability of ADC target expression, paired comparisons were performed between primary tumors and their matched pathological components. For HER2 and Trop-2, the IHC expression intensity was significantly higher in invasive primary tumors compared to their matched CIS components (P = 0.034 and P = 0.039, respectively), suggesting a potential upregulation during the transition from CIS to invasive carcinoma. Regarding the comparison between primary tumors and lymph node metastases, no significant disparities were observed for any of the three targets (all P > 0.05), indicating relative stability during lymphatic spread. Although Nectin-4 expression did not reach statistical significance in paired comparisons (primary vs. CIS, P = 0.163; primary vs. lymph node metastasis, P = 0.305), a numerical trend towards lower expression in CIS and metastatic lesions was noted compared to the primary tumors. Detailed paired data are summarized in **Table 4**.

**Table 4:** Within-patient paired comparisons of IHC intensity.

Marker	Paired comparison	n (pairs)	Negative/positive/ties*	vZ	P value	Effect size r^†^
HER2	Tumor vs CIS	8	5/0/3	-2.121	**0.034**	0.750
HER2	Tumor vs LN metastasis	12	5/3/4	-1.294	0.196	0.374
Trop2	Tumor vs CIS	8	5/0/3	-2.060	**0.039**	0.728
Trop2	Tumor vs LN metastasis	12	7/3/2	-1.503	0.133	0.434
Nectin4	Tumor vs CIS	8	4/2/2	-1.394	0.163	0.493
Nectin4	Tumor vs LN metastasis	12	6/2/4	-1.027	0.305	0.296

*Negative indicates CIS/LN intensity < tumor intensity; Positive indicates CIS/LN intensity > tumor intensity; Ties indicate no change.

^†^Effect size r = |Z|/√n.

CIS vs LN metastasis paired testing was not performed due to insufficient paired specimens.

Bold values indicate statistical significance (p < 0.05).

#### 3.1.3 Expression divergence between the upper and lower urinary tract

The expression of the three ADC targets was compared between the upper urinary tract and the lower urinary tract. The Mann–Whitney U test revealed that Trop-2 expression was significantly higher in the lower urinary tract than in the upper urinary tract (U = 617.500, Z = -2.032, P = 0.042), with a calculated effect size of r = 0.187. Conversely, no significant differences were observed in the expression levels of HER2 or Nectin-4 between these two anatomical regions (both P > 0.05). Detailed comparative data across anatomical sites are summarized in **Table 5**.

**Table 5:** Upper vs lower urinary tract differences.

Marker	Upper n	Lower n	Mean rank (Upper)	Mean rank (Lower)	U	Z	P value	Effect size r*
HER2	17	101	57.56	59.83	825.500	-0.267	0.790	0.025
Trop2	17	101	45.32	61.89	617.500	-2.032	**0.042**	0.187
Nectin4	17	101	55.91	60.10	797.500	-0.490	0.624	0.045

*Effect size r = |Z|/√N.

Bold values indicate statistical significance (p < 0.05).

#### 3.1.4 Correlation between target expression and clinicopathological factors

##### 3.1.4.1 Univariate exploratory analysis

Univariate exploratory analysis was performed using the IHC expression intensity scores (0–3+) of HER2, Trop-2, and Nectin-4 in tumor tissues as outcome variables. The Mann–Whitney U test was applied for dichotomous clinicopathological variables, and Spearman’s rank correlation analysis was utilized for continuous variables (age and maximum tumor diameter).Regarding dichotomous variables, histological grade (high vs. low) was significantly associated with HER2 expression intensity, with the high-grade group exhibiting substantially higher expression levels (mean rank: 64.70 vs. 47.16; Z = -2.761, P = 0.006). Furthermore, the presence of concurrent CIS was significantly correlated with Trop-2 expression intensity; patients in the CIS-present group demonstrated higher Trop-2 expression compared to those in the CIS-absent group (mean rank: 88.00 vs. 58.27; Z = -2.711, P = 0.007). No statistically significant associations were observed between other variables—including metastasis, sex, LVI, and recurrence—and the expression intensities of the three targets (all P > 0.05; **Table 6A**).In the correlation analysis for continuous variables, tumor size (maximum diameter) demonstrated a weak negative correlation with Nectin-4 expression intensity (Spearman ρ = -0.246, P = 0.007), suggesting that Nectin-4 expression tends to decrease as tumor size increases. Age was not significantly correlated with the expression levels of any of the three targets (all P > 0.05; **Table 6B**).

**Table 6A:** Univariate associations between clinicopathological factors and tumor IHC intensity.

Variable (group A vs group B)	HER2 mean rank A/B (nA/nB)	Z	P	Trop2 mean rank A/B (nA/nB)	Z	P	Nectin4 mean rank A/B (nA/nB)	Z	P
Metastasis (Yes vs No)	65.00/59.86 (15/105)	-0.565	0.572	59.80/60.60 (15/105)	-0.092	0.927	52.93/61.58 (15/105)	-0.944	0.345
Sex (Female vs Male)	61.25/60.33 (22/98)	-0.118	0.906	58.23/61.01 (22/98)	-0.373	0.709	69.32/58.52 (22/98)	-1.379	0.168
Pathological grade (High vs Low)	64.70/47.16 (79/38)	-2.761	**0.006**	61.70/53.38 (79/38)	-1.37	0.171	56.20/64.82 (79/38)	-1.347	0.178
Tumor thrombus (Yes vs No)	56.74/61.21 (19/101)	-0.542	0.588	56.84/61.19 (19/101)	-0.549	0.583	49.34/62.60 (19/101)	-1.597	0.11
Concomitant CIS (Yes vs No)	73.17/59.47 (9/111)	-1.199	0.231	88.00/58.27 (9/111)	-2.711	**0.007**	76.50/59.20 (9/111)	-1.503	0.133
Recurrence (Yes vs No)	65.11/60.13 (9/111)	-0.436	0.663	65.67/60.08 (9/111)	-0.509	0.611	72.33/59.54 (9/111)	-1.112	0.266

Bold values indicate statistical significance (p < 0.05).

**Table 6B:** Spearman correlations between continuous variables and tumor IHC intensity.

Continuous variable	HER2 ρ	P	Trop2 ρ	P	Nectin4 ρ	P
Age (years)	0.138	0.133	-0.016	0.858	-0.074	0.424
Tumor maximum diameter (mm)	0.109	0.24	0.006	0.948	-0.246	**0.007**

Bold values indicate statistical significance (p < 0.05).

##### 3.1.4.2 Multivariable logistic regression analysis

To identify independent predictors of target expression, multivariable regression models were constructed. For HER2 and Trop-2, ordinal logistic regression was employed using the 0–3+ expression intensity scores as ordinal outcomes. The proportional odds assumption was verified using the test of parallel lines. For Nectin-4, as the proportional odds assumption was violated in the ordinal model (P < 0.05), it was converted into a binary outcome (“high expression” [2–3+] vs. “low expression” [0–1+]) and analyzed using multivariable binary logistic regression. The detailed results are summarized in **Table 7**.

**Table 7:** Multivariable models for ADC-related targets.

Panel/Outcome	Predictor (coding)	aOR/OR (95% CI)	P value
A. HER2 intensity (0–3) Ordinal logistic (proportional odds)	Clinical stage (per 1-unit increase)	0.884 (0.630–1.242)	0.479
	Recurrence (Yes=1 vs No=0)	0.860 (0.222–3.326)	0.827
	Pathological grade (1 vs 0)	**3.162 (1.323–7.554)**	**0.010**
	Tumor location (Upper tract=1 vs Lower tract=0)	0.775 (0.294–2.042)	0.606
B. Trop2 intensity (0–3) Ordinal logistic (proportional odds)	Age (per 1-year increase)	0.991 (0.957–1.026)	0.610
	Pathological grade (1 vs 0)	**2.702 (1.175–6.221)**	**0.019**
	Metastasis (Yes=1 vs No=0)	1.528 (0.458–5.094)	0.490
	Muscle invasion (Yes=1 vs No=0)	**0.267 (0.106–0.670)**	**0.005**
C. Nectin4 high expression (2–3 vs 0–1) Binary logistic	Tumor thrombus (Yes=1 vs No=0)	0.417 (0.131–1.327)	0.139
	Concomitant CIS (Yes=1 vs No=0)	**5.869 (1.104–31.204)**	**0.038**
	Recurrence (Yes=1 vs No=0)	3.537 (0.793–15.779)	0.098
	Sex (Male=1 vs Female=0)	**0.292 (0.107–0.794)**	**0.016**
	Age (per 1-year increase)	0.997 (0.960–1.037)	0.897

Binary coding: upper tract=1/lower=0; thrombus=1/0; CIS = 1/0; recurrence=1/0; metastasis=1/0; male=1/female=0. Panels A–B: proportional-odds ordinal logistic regression for tumor IHC intensity (0–3). Panel C: binary logistic regression for Nectin4 high expression (2–3 vs 0–1).

Bold values indicate statistical significance (p < 0.05).

For HER2, pathological grade was independently associated with expression intensity (adjusted odds ratio [aOR] = 3.162; 95% CI: 1.323–7.554; P = 0.010). Other factors, including clinical stage, recurrence, and anatomical location (UTUC vs. lower tract UC), did not reach statistical significance. The test of parallel lines (P = 0.265) confirmed the validity of the proportional odds assumption for this model.

For Trop-2, high histological grade was significantly associated with higher expression levels (aOR = 2.702, P = 0.019), while muscle-invasive disease was correlated with lower Trop-2 expression (aOR = 0.267, P = 0.005). The proportional odds assumption was satisfied (P = 0.505).For Nectin-4, the binary logistic regression revealed that concurrent CIS was positively associated with high expression (OR = 5.869; 95% CI: 1.104–31.204; P = 0.038). Interestingly, male sex was negatively associated with Nectin-4 high expression (OR = 0.292; 95% CI: 0.107–0.794; P = 0.016).

### 3.2 Secondary outcomes

#### 3.2.1 Co-high expression landscape of ADC targets: UpSet plot analysis

To further evaluate the distribution of potential therapeutic beneficiaries, the co-high expression patterns of Nectin-4, HER2, and Trop-2 were analyzed using an UpSet plot. Among the 118 specimens, 107 (90.7%) exhibited high expression (IHC ≥ 2+) of at least one target. In terms of overall prevalence, Trop-2 was the most frequent high-expressing target (n = 89), followed by HER2 (n = 53) and Nectin-4 (n = 51).

Single-target high expression was identified in 40 cases (33.9%), with “Trop-2 only” being the most common phenotype (n = 27), which significantly outnumbered “Nectin-4 only” (n = 7) and “HER2 only” (n = 6). Notably, 67 patients (56.8%) demonstrated concurrent high expression of two or three targets. The most prevalent double-high combinations were HER2 + Trop-2 (n = 23) and Nectin-4 + Trop-2 (n = 20), whereas the Nectin-4 + HER2 combination was relatively rare (n = 5). A “triple-high” phenotype (concurrent high expression of all three targets) was identified in 19 cases (16.1%). Conversely, a “triple-low” subgroup—defined as failing to reach the high-expression threshold (IHC 0/1+) for any of the three markers—accounted for only 9.3% (n = 11) of the cohort.

**Figure 4:**
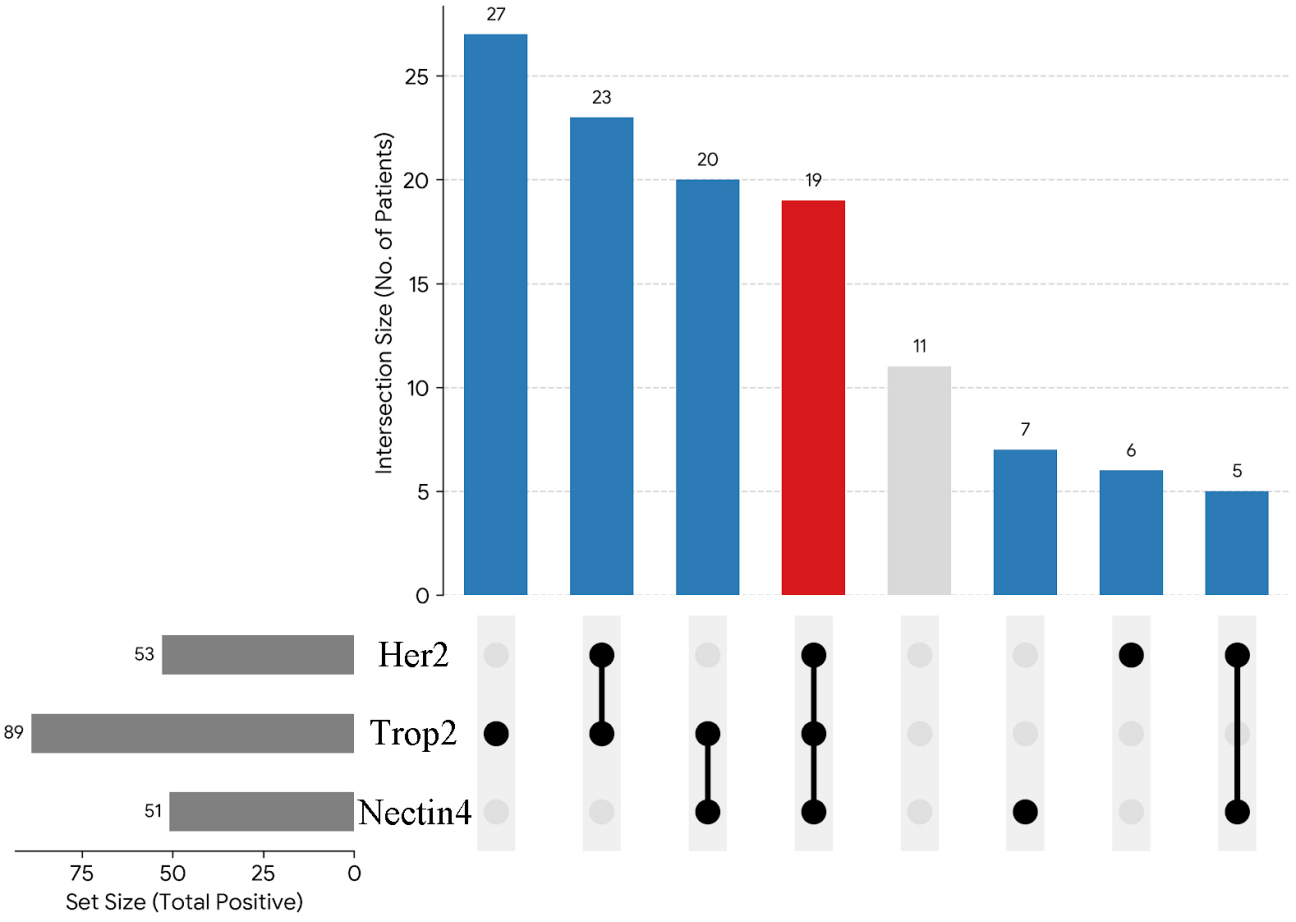
UpSet plot characterizing the co-high expression landscape of ADC targets. Horizontal bars (bottom left) represent the total number of cases with high expression (defined as IHC 2+ or 3+) for each individual target across the cohort (n=118). Vertical bars (top) indicate the number of patients in each unique intersection (specific combination of high-expression targets). Filled black circles and connecting lines denote the specific targets included in a given intersection. The triple-high-expression intersection is highlighted in red, while the triple-low-expression/negative (IHC 0 or 1+) intersection is shown in gray.

Consistent with the pharmacological mechanism of ADCs, which utilize surface proteins as molecular anchors for cytotoxic payload delivery rather than relying solely on oncogenic driver inhibition, “high expression” in this study was defined as an IHC score ≥ 2 +. Consequently, in alignment with the clinical eligibility criteria of the RC48-C005/C009 trials, HER2 status was determined by IHC alone without additional fluorescence *in situ* hybridization (FISH) assessment (**Figure 4**).

#### 3.2.2 Association between δ scores and clinical factors

In cases where paired IHC scores for both tumor and adjacent normal tissues were available (n = 60), a Δ score was calculated (Δ score = tumor score − adjacent normal score) to quantify the expression increment in malignant versus benign tissues. Overall, the median Δ scores for HER2 and Trop-2 were 0 (IQR: 0–1), while the median Δ score for Nectin-4 was 1 (IQR: 0–1).In comparisons of dichotomous variables, ΔTrop-2 demonstrated significant associations with LVI status (P = 0.016), metastatic status (P = 0.007), and muscle invasion (P = 0.049). ΔNectin-4 was significantly correlated with the presence of concurrent CIS (P = 0.016) and sex (P = 0.030), while ΔHER2 showed an association with sex (P = 0.010). No significant differences were observed for other factors, including anatomical location (UTUC vs. lower tract UC), recurrence, or histological grade.

Spearman correlation analysis revealed that maximum tumor diameter was positively correlated with ΔTrop-2 (ρ = 0.325, P = 0.011) and negatively correlated with ΔNectin-4 (ρ = -0.331, P = 0.010). However, age and clinical stage showed no statistically significant correlations with any of the Δ scores (**Table 8**).

**Table 8:** Association of ΔIHC scores with clinicopathological features.

Clinicopathological factors	Coding/statistics	ρ (ΔHER2)	P	ρ (ΔTrop2)	P	ρ (ΔTrop2)	P
Categorical Variables							
Upper vs Lower tract	Upper/Lower	—	0.214	—	0.946	—	0.454
Sex	Male/Female	—	0.01	—	0.501	—	0.03
Tumor thrombus	Yes/No	—	0.377	—	0.016	—	0.344
Muscle invasion	Yes/No	—	0.824	—	0.049	—	0.361
Concomitant CIS	Yes/No	—	0.866	—	0.598	—	0.016
Metastasis	Yes/No	—	0.805	—	0.007	—	0.27
Pathological grade	High/Low	—	0.343	—	0.109	—	0.053
Recurrence	Yes/No	—	0.149	—	0.918	—	0.547
Continuous Variables							
Age	ρ	0.156	0.234	-0.183	0.161	-0.169	0.197
Tumor maximum diameter	ρ	0.216	0.098	0.325	0.011	-0.331	0.01
Clinical stage	ρ	-0.05	0.702	0.244	0.061	-0.16	0.221

Δscore = Tumor IHC score − Adjacent normal IHC score. CIS, concomitant carcinoma *in situ*. For categorical variables, P-values were calculated using the Mann-Whitney U test. For continuous variables, ρ represents the Spearman correlation coefficient.

## 4 Discussion

This study provides a systematic evaluation of the horizontal expression of Nectin-4, HER2, and Trop-2 across the entire urinary tract and diverse pathological contexts within a single cohort. Our findings confirm that expression intensities and positivity rates are significantly higher in malignant tissues than in matched adjacent normal tissues, underscoring a favorable therapeutic window for target-directed ADCs. Furthermore, our analysis of co-expression patterns revealed that 90.7% of patients with UC expressed at least one of these targets, while over 56.8% demonstrated multi-target co-expression. This extensive overlap highlights a high therapeutic redundancy, suggesting that when resistance to an primary agent occurs, sequentially switching to Trop-2- or HER2-targeted ADCs may serve as a pivotal salvage strategy.

A particularly significant finding with clinical implications in this study was the substantial downregulation of target expression in metastatic lesions. Specifically, the 66.67% negativity rate of Nectin-4 in lymph node metastases, coupled with varying degrees of attenuation in HER2 and Trop-2, challenges the conventional paradigm of “guiding systemic therapy based on primary tumor status”, which is consistent with the results of Klumper et al. (13) This heterogeneity may stem from “clonal selection” during distant migration, wherein sub-clones with lower target expression may possess enhanced epithelial-mesenchymal transition capabilities or immune evasion potential (14). Consequently, over-reliance on archived primary tumor specimens in the precision treatment of advanced UC may lead to suboptimal ADC selection. Our data strongly advocate for the implementation of “multi-site dynamic biopsies” in patients with relapsed or progressive disease to mitigate the risk of treatment failure mediated by antigen loss.

Spatial heterogeneity remains a formidable challenge in precision oncology. Our analysis of matched specimens reveals a marked upregulation of HER2 and Trop-2 during the transition from carcinoma *in situ* to invasive carcinoma. This trend suggests that the overexpression of these two targets may facilitate the critical biological shifts required for tumors to bypass growth restrictions and achieve invasive potential (15).

Historically, upper tract urothelial carcinoma and urothelial carcinoma of the bladder have often been managed as a homogenized entity in clinical practice. However, our findings demonstrate significantly higher Trop-2 expression in the lower urinary tract, implying a broader therapeutic reach for Trop-2-targeted ADCs in UCB patients (16). Conversely, the consistent distribution of HER2 and Nectin-4 supports their broad-spectrum applicability across the entire urinary tract (17, 18). These findings enable “anatomically-informed” drug selection, allowing clinicians to leverage the localized superiority of certain targets when multiple markers are present.

Furthermore, multivariable ordinal logistic regression established pathological grade as an independent predictor for HER2 and Trop-2 expression intensity. High-grade tumors, characterized by increased genomic instability, tend to overexpress these targets, providing a precise rationale for treating refractory, high-grade UC. Interestingly, Nectin-4 expression correlated positively with the presence of CIS but showed a weak negative correlation with maximum tumor diameter. This divergence may reflect the dominant role of Nectin-4 during early-stage oncogenesis or within specific clonal sub-populations, whereas its expression may dwindle due to “clonal dilution” or active downregulation as tumor volume expands and clonal evolution proceeds, which is in line with the views of Aggen et al. (13, 19).

Our study innovatively introduces the Δ score, which filters out inter-individual baseline variations in urothelial expression and more accurately reflects the “net gain” of target proteins during malignant transformation. Notably, ΔTrop-2 was significantly associated with muscle invasion, LVI, and metastatic status. This finding hints at a potential molecular link between Trop-2 overexpression and the vascular invasive potential of UC, positioning the net increment of Trop-2 as a promising biomarker for assessing tumor aggressiveness.

Furthermore, our results provide several clinical considerations for personalized ADC selection. First, the observed shifts in expression levels across disease stages suggest that the optimal ADC target may evolve throughout the disease course, necessitating a sequential treatment strategy. Second, given the predominant “moderate abundance” (IHC 2+) and limited “high abundance” (IHC 3+) observed in our cohort—coupled with the downregulation in metastases—careful consideration of ADC payload characteristics is essential. While the efficacy of Enfortumab Vedotin, which carries an MMAE payload with a moderate bystander effect, may be challenged by antigen loss in metastatic sites, agents like Trastuzumab Deruxtecan (T-DXd) might offer superior clinical robustness. The potent membrane permeability and robust bystander killing capacity of the Dxd payload may more effectively overcome the “target abundance ceiling” and intratumoral heterogeneity identified in our study (20, 21). Finally, the high coverage observed—with the vast majority of patients harboring at least one positive target—supports the future exploration of dual-target ADCs or the sequential use of different ADCs to circumvent acquired resistance mediated by antigen loss.

Study Limitations: Despite the comprehensive scope of this study, several limitations warrant acknowledgment. First, the retrospective nature and the relatively limited sample size of distant metastatic specimens may constrain the generalizability of our conclusions regarding late-stage spatial heterogeneity. Second, while immunohistochemistry remains the established clinical standard for protein assessment, it provides a snapshot of protein abundance that does not account for the potential influences of gene amplification or mRNA-level regulation on ADC efficacy. Third, the novel Δ-score is currently an unvalidated metric; although a high Δ-score may mathematically reflect low baseline expression in normal tissue rather than intrinsic tumor aggressiveness, this divergence clinically signifies an optimal therapeutic window and empirically correlated with aggressive phenotypes in our cohort. Finally, this study lacked direct correlation with clinical outcomes, such as objective response rates or survival data. Future prospective investigations integrating longitudinal clinical follow-up and multi-omics profiling are required to further validate the predictive value of these target-based therapeutic strategies and the Δ-score in the era of precision oncology.

## 5 Conclusion

This study systematically maps the expression landscape and spatial heterogeneity of Nectin-4, HER2, and Trop-2 in urothelial carcinoma. While 90.7% of patients exhibited actionable expression of at least one target and over half showed multi-target co-expression, significant target fluctuations across anatomical sites and during metastatic progression underscore the inherent limitations of single-site biopsies. Additionally, the novel Δ-score provides a potential exploratory framework to quantify net antigenic gain and its correlation with aggressive phenotypes. Collectively, our findings emphasize the necessity of integrating anatomical origin, stage evolution, and metastatic dynamics into clinical sequencing, providing a strong theoretical foundation for precision, sequential ADC therapeutic strategies in advanced UC.

## Data Availability

The original contributions presented in the study are included in the article/supplementary material. Further inquiries can be directed to the corresponding author.
